# Predictive Value of Skeletal Muscle Mass in Recurrent/Metastatic Head and Neck Squamous Cell Carcinoma Patients Treated With Immune Checkpoint Inhibitors

**DOI:** 10.3389/fonc.2021.699668

**Published:** 2021-06-25

**Authors:** Lorena Arribas, Maria Plana, Miren Taberna, Maria Sospedra, Noelia Vilariño, Marc Oliva, Natalia Pallarés, Ana Regina González Tampán, Luis Miguel Del Rio, Ricard Mesia, Vickie Baracos

**Affiliations:** ^1^ Clinical Nutrition Unit, Catalan Institute of Oncology (ICO), L’Hospitalet de Llobregat, Barcelona, Spain; ^2^ Bellvitge Biomedical Research Institute (IDIBELL), L’Hospitalet de Llobregat, Barcelona, Spain; ^3^ Head and Neck Cancer Unit, Bellvitge University Hospital, Catalan Institute of Oncology (ICO), L’Hospitalet de Llobregat, Barcelona, Spain; ^4^ University of Barcelona, Barcelona, Spain; ^5^ Medical Oncology Department, Catalan Institute of Oncology (ICO), ONCOBELL, L’Hospitalet de Llobregat, Barcelona, Spain; ^6^ Unitat de Nutrició Clínica i Dietètica, Hospital Universitari Germans Trias i Pujol (HUGTiP), Barcelona, Spain; ^7^ Unitat de Bioestadística (UBiDi), Bellvitge University Hospital, L’Hospitalet de Llobregat, Barcelona, Spain; ^8^ Grupo Ascires, CETIR Grupo Médico, Barcelona, Spain; ^9^ Medical Oncology Department, Catalan Institute of Oncology (ICO)-Badalona, B-ARGO group, Barcelona, Spain; ^10^ Division of Palliative Care Medicine, Department of Oncology, University of Alberta, Cross Cancer Institute, Edmonton, AB, Canada

**Keywords:** head and neck (H&N) cancer, body composition, muscle mass, immune checkpoint inhibitors, sarcopenia, immune-related adverse events (irAE)

## Abstract

**Background:**

Reduced muscle mass has been associated with increased treatment complications in several tumor types. We evaluated the impact of skeletal muscle index (SMI) on prognosis and immune-related adverse events (IrAEs) in a cohort of recurrent/metastatic (R/M) head and neck squamous cell carcinoma (HNSCC) treated with immune checkpoints inhibitors (ICI).

**Methods:**

A single-institutional, retrospective study was performed including 61 consecutive patients of R/M HNSCC diagnosed between July 2015 and December 2018. SMI was quantified using a CT scan at L3 to evaluate body composition. Median baseline SMI was used to dichotomize patients in low and high SMI. Kaplan-Meier estimations were used to detect overall survival (OS) and progression-free survival (PFS). Toxicity was recorded using Common Terminology Criteria for Adverse Event v4.3.

**Results:**

Patients were 52 men (85.2%) with mean of age 57.7 years (SD 9.62), mainly oral cavity (n = 21; 34.4%). Low SMI was an independent factor for OS in the univariate (HR, 2.06; 95% CI, 1.14–3.73, p = 0.017) and multivariate Cox analyses (HR, 2.99; 95% CI, 1.29–6.94; p = 0.011). PFS was also reduced in patients with low SMI (PFS HR, 1.84; 95% CI, 1.08–3.12; p = 0.025). IrAEs occurred in 29 (47.5%) patients. There was no association between low SMI and IrAEs at any grade (OR, 0.56; 95% CI, 0.20–1.54; p = 0.261). However, grades 3 to 4 IrAEs were developed in seven patients of whom three had low SMI.

**Conclusions:**

Low SMI before ICI treatment in R/M HNSCC patients had a negative impact on OS and PFS. Further prospective research is needed to confirm the role of body composition as a predictive biomarker in ICI treatment.

## Introduction

Among patients with head and neck squamous cell carcinoma (HNSCC), between 5% and 10% are diagnosed with metastatic disease. Additionally, despite aggressive multimodal strategies, about 60% of patients treated with radical intention for a locally advanced disease will eventually recur (1). Until the introduction of immunotherapy agents, the median survival was 10.1 months, with an 82% rate of grades 3 to 4 adverse events using the historic standard first-line EXTREME (combining platinum and 5-fluorouracil (5-FU) and cetuximab) (2). Patients with progressive disease after platinum-based chemotherapy have a poor prognosis with a 1-year survival under 5% (3). Hereby, there is an urgent need for improved therapy in the recurrent and metastatic (R/M) population.

Targeting the programmed cell death (ligand)-1 (PD-(L)1) pathway has shown significant activity, and improved overall survival (OS) in patients with previously treated R/M HNSCC, associated with fewer grades 3 or 4 toxicities than standard therapy (4, 5). These results have led to approval of two anti-PD1 agents (pembrolizumab and nivolumab) as second-line treatment for patients with R/M HNSCC who experience disease progression on or after a platinum-based therapy (6, 7). More recently, pembrolizumab has been approved in the first-line setting, alone or in combination with chemotherapy (8). Despite improving the results compared with older strategies, approximately 70% of patients do not benefit from immune checkpoint inhibitors (ICI) as they have progression as the best response, enhancing the need for predictive biomarkers (6, 8).

Patients with R/M HNSCC are at an increased nutritional risk, and malnutrition has been shown to be an independent indicator of prognosis in cancer patients (9). The nutritional deterioration of HNSCC patients is often present from diagnosis and worsens throughout onco-specific treatments (10). This deterioration does not only occur exclusively at the expense of weight alone but also because the loss of muscle mass has been shown to associate with prognosis and complications (10, 11).

Sarcopenia, defined as a reduced skeletal muscle mass that reduce muscle function, is noted in geriatric populations (12). Reduced muscle mass is also prominent in patients at any age with different chronic diseases, including cancer. This is also termed sarcopenia, and it is typically classified in relation to the risk of disease-specific outcomes, such as mortality, surgical complications, or cancer treatment (13). Sarcopenia has been reported to have a significant impact on both OS and complications in cancer patients undergoing onco-specific treatment and/or surgery (14). These results have also been described in head and neck cancer patients (15–18). Although some studies have revealed that low muscle mass may also have a role in the oncological outcomes in patients with melanoma (19, 20) or lung cancer (21, 22) treated with ICI, as far as we know, there are no current studies evaluating the impact of low muscle mass in R/M HNSCC undergoing these therapies.

We aim to evaluate the muscle mass as a predictive biomarker of OS and progression-free survival (PFS) in patients diagnosed with R/M HNSCC treated with ICI. A secondary analysis focused on the association of muscle mass on the onset of immune-related adverse events (IrAES).

## Materials and Methods

### Population and Study Design

This longitudinal retrospective single-center study was approved by the local ethics committee for clinical research (PR302/18). All patients provided written informed consent. Patients diagnosed with R/M HNSCC treated with ICI, regardless of treatment line, from July 2015 and December 2018 at the Catalan Institute of Oncology, were evaluated. Patients were eligible if they had R/M HNSCC and were treated with ICI including anti-PD1 or anti-PDL1 alone or in combination with other ICI (such as anti-CTLA4) or chemotherapy and had a staging full-body computed tomography (CT) scan as part of their pre-treatment procedure (within 10 days prior to the introduction of ICI) and at evaluation of tumor response according to RECIST criteria, version 1.1 (23).

Clinical data included age, sex, smoking status, alcohol consumption, Eastern Cooperative Oncology Group Performance Status (ECOG-PS), TNM on Cancer (7th edition) (24), primary tumor site, treatment line for R/M disease, type of recurrence prior ICI, and response. Those patients, who had received the last dose of platinum 6 months before of initiation of ICI, were classified as platinum-refractory. Additional information regarding IrAEs according to the CTCAE version 4.3 2009 (25) and vital status were also collected from medical records.

Nutritional data were collected at baseline (before starting treatment). These data included body mass index (BMI calculated as [(weight (kg)/height (m^2^)], serum albumin levels, and type of nutritional intervention if any.

### Image Analysis

All treatment images were selected by a trained researcher to ensure they correspond to the same vertebra landmarks to allow a proper comparison of body composition. Values were obtained by a single observer blinded to the patients’ data.

Images were accessed from the axial cross-sectional CT as all patients had an abdominal CT scan as part of their routine care. The third lumbar (L3) vertebra was chosen on the axial cross-section CT component of the full-body CT scans as the reference point, based on previous reports with this level to calculate the skeletal muscle index (SMI) (26, 27) using SliceOmatic^©^ software (v5.0 Rev 8, Tomovision, Montreal, Quebec, Canada). Regional analysis at L3 strongly predicted whole-body fat and fat-free mass (r=0.86-0.94; p < 0.001) (26). Muscle cross-sectional area (CSA) was quantified within a Hounsfield unit (HU) range from -29 to +150HU and then normalized for height to report as SMI (cm^2^/m^2^). Sarcopenia was defined according to Martin L et al. (28) using specific SMI cut points for advanced cancer patients. CSA of adipose tissue were determined using tissue-specific HU range defined at this level (29).

### Statistical Analysis

To define cohort characteristics, categorical variables were presented as the number of cases and percentages, whereas continuous variables were presented as the mean and standard deviation (SD) or median and interquartile range (IQR). Median baseline SMI was used to dichotomize patients in two groups: low SMI (patients with baseline SMI lower than median baseline SMI) or high SMI (patients with baseline SMI equal or higher than median baseline SMI).

It was planned to test for the effect of baseline SMI on survival. Time between treatment initiation and disease progression or death from any cause (PFS) and time between treatment initiation and death from any cause (OS) was assessed using the Kaplan-Meier estimator. One-year OS rate and 1-year PFS rate were also analyzed. The Cox proportional hazards model was used to perform univariate and multivariate survival analyses, which are reported as the hazard ratio (HR) and 95% confidence interval. Covariates with a p-value lower than 0.1 in the univariate model were included in the multivariate models. The proportionality of risks in the Cox model was verified using the Schoenfeld residuals.

To evaluate the effect of baseline variables in the development of toxicity, logistic regression models were used. Odds ratios and their corresponding 95% confidence intervals were derived from both univariate and multivariate models.

Statistical significance was set at a probability level ≤0.05. The statistical package used to treat the data and perform the statistical analysis was R software version 3.5.

## Results

### Patient Characteristics

**Table 1** shows baseline demographic and clinical characteristics of the 61 patients included in the analysis. Most patients were male (n = 52, 85.2%) with a mean age of 57.7 years (SD 9.62). Tumor location was mainly oral cavity (n = 21; 34.4%). Most of patients recurred with locoregional plus metastatic disease (n = 28; 45.9%). Four (6.5%) patients received ICI as the first treatment and 59% (n = 36) were platinum refractory.

**Table 1:** Patient baseline characteristics (overall and according to low *vs* high skeletal muscle index) (SMI) (n=61).

	Overall (n=61)	Low SMI (n=30)	High SMI (n=31)	p-overall
Age, years				
Mean (SD)	57.7 (9.62)	55.1 (9.93)	60.3 (8.73)	0.035
Median (range)	59.0 (23-78)	55.9 (23-70)	61.3 (35-78)	0.753
Male, n (%)	52 (85.2)	24 (80.0)	28 (90.3)	0.301
Smoking status, n (%)				0.394
Current	28 (45.9)	16 (53.3)	12 (38.7)	
Former*	26 (42.6)	12 (40.0)	14 (45.2)	
Never	7 (11.5)	2 (6.7)	5 (16.1)	
Alcohol consumption^#^, n (%)				
Yes	36 (59.0)	19 (63.3)	17 (54.8)	0.699
Location, n (%)				0.283
Oral cavity	21 (34.4)	11 (36.7)	10 (32.3)	
Hypopharynx	8 (13.1)	5 (16.7)	3 (9.7)	
Larynx	19 (31.3)	6 (20.0)	13 (41.9)	
Oropharynx**	13 (21.3)	8 (26.7)	5 (16.1)	
Type of recurrence				0.700
Locoregional	23 (37.7)	10 (33.3)	13 (41.9)	
Distance	10 (16.4)	6 (20.0)	4 (12.9)	
Locoregional + distance	28 (45.9)	14 (46.7)	14 (45.2)	
Line of therapy, n (%)				0.865
First	22 (36.1)	10 (33.3)	12 (38.7)	
Second or above	39 (63.9)	20 (66.7)	19 (61.3)	
Type of ICI therapy, n (%)				0.786
AntiPD1	8 (13.1)	5 (16.7)	3 (9.7)	
AntiPD1+virus	1 (1.64)	0 (0.0)	1 (3.3)	
AntiPD1 + chemotherapy	3 (4.92)	2 (6.7)	1 (3.2)	
AntiPDL1	12 (19.7)	5 (16.7)	7 (22.6)	
AntiPDL1+antiCTLA4	22 (36.1)	11 (36.7)	11 (35.5)	
AntiPDL1+IOA	15 (24.6)	9 (29.0)	6 (20.0)	
ECOG-PS, n (%)				0.363
0	1 (1.64)	0 (0.0)	1 (3.2)	
1	58 (95.1)	30 (100)	28 (90.3)	
2	2 (3.28)	0 (0.0)	2 (6.5)	
Platinum within 6 months of ICI,n (%)	36 (59.0)	18 (60.0)	18 (58.1)	1.000
Weight, kg				
Mean (SD)	67.3 (15.0)	59.5 (10.9)	74.9 (14.7)	<0.001
Median [Q1; Q3]	65.2 [54.3;79.0]	58.8 [51.0;65.6]	77.5 [64.3;84.5]	<0.001
BMI, kg/m^2^				
Mean (SD)	23.8 (4.56)	21.3 (3.33)	26.2 (4.32)	<0.001
Median (range)	23.6 (15.8-34.7)	21.7 (15.8-27.6)	26.8 (17.7-34.6)	<0.001
BMI categorized, kg/m^2^				0.001
Underweight (<18.5)	9 (14.8)	7 (23.3)	2 (6.5)	
Normal (18.5 – 25)	26 (42.6)	17 (56.7)	9 (29.0)	
Overweight /obese (>25)	26 (42.6)	6 (20.0)	20 (64.5)	
Albumin, g/L				
Mean (SD)	42.9 (60.3)	43.0 (3.24)	42.9 (7.86)	0.933
Median [Q1; Q3]	44.0 [41.0;46.0]	43.0 [41.0;45.0]	44.0 [42.0;46.5]	0.302
SMI, cm^2^/m^2^				
Mean (SD)	43.6 (7.75)	37.2 (3.14)	49.8 (5.44)	<0.001
Median [Q1; Q3]	42.0 [37.5;48.6]	37.5 [35.2;39.6]	48.6 [46.1;53.0]	<0.001
TATI, cm^2^/m^2^				
Mean (SD)	91.4 (53.3)	71.7 (44.5)	111 (54.7)	0.003
Median [Q1; Q3]	98.8 [49.4;118]	70.8 [31.5;101]	112 [82.0;148]	0.004

*Ex-smoker defined as no cigarettes for more than 6 months before diagnosis.

^#^Alcohol consumption defined as sustained heavy drinker (≥4 drinks per week in women and ≥5 drinks per week in men). Includes active and former drinkers.

ICI, immune checkpoint inhibitor; ECOG-PS, Eastern Cooperative Oncology Group Performance Status; BMI, body mass index; SMI, skeletal muscle index; TATI, total adipose tissue index; IOA, immuno-oncology agent.

**3 of them HPV-related.

At baseline, the mean BMI was 23.8 kg/m^2^ (SD 4.56); underweight (BMI ≤18.5) was present in 9 (14.8%) patients and 26 (42.6%) were overweight (BMI ≥25) or obese (BMI ≥30). Median SMI was 42.0 cm^2^/m^2^ (IQR 37.5; 48.6) and was used to classify patients between high and low SMI. Nutritional support was required in 34 (55.7%) patients, 15 (44.1%) of them needed a tube feeding. Two thirds (n=41, 67.2%) of the patients were sarcopenic according to previously published cut points (30) and three of them were also obese (**Table 1S**, **Supporting Information**).

Significant differences were identified for patients with low *vs* high SMI in mean age (p = 0.035), baseline weight (p < 0.001), BMI (P < 0.001), and total adipose tissue (p = 0.003). Patients with high SMI were older, heavier, and with higher BMI. No other significant differences between patients with low and high SMI at baseline were found.

### Effects of SMI in Overall Survival (OS) and Progression Free Survival (PFS)

Median follow-up time was 9 months (range, 3.6–21.3). Up to a third of patients (n=16; 26.2%) were alive at last follow-up. The median time to death was 4.3 months (range, 2.3–10.9). **Table 2** summarized univariate and multivariate analyses of OS, PFS, 1-year survival, and 1-year PFS.

**Table 2:** Univariate and multivariate analysis examining OS, one-year survival, global PFS and one-year PFS in association with skeletal muscle index (SMI) (n=61).

	SURVIVAL						PROGRESION FREE SURVIVAL					
	Overall survival			1-year survival			Global PFS			1-year PFS		
Univariate analysis	*HR*	*95% IC*	*P value*	*HR*	*95% IC*	*P value*	*HR*	*95% IC*	*P value*	*HR*	*95% IC*	*P value*
BMI	0.97	0.91;1.04	0.432	0.96	0.89;1.03	0.232	0.96	0.91;1.02	0.237	0.96	0.90;1.03	0.235
Age	0.98	0.96;1.01	0.177	0.98	0.95;1.00	0.077	0.96	0.94;0.99	0.002	0.96	0.94;0.99	0.002
Serum albumin	0.97	0.93;1.00	0.056	0.97	0.93;1.01	0.112	0.95	0.91;0.99	0.027	0.95	0.90;0.99	0.024
Low skeletal muscle index	2.06	1.14;3.73	0.017	2.64	1.33;5.23	0.005	1.84	1.08;3.12	0.025	1.83	1.01;3.23	0.036
Platinum-refractory	1.76	0.94;3.28	0.075	1.85	0.91;3.78	0.089	3.04	1.67;5.56	<0.001	2.95	1.57;5.55	0.001
Type of recurrence												
Distance	0.86	0.35;2.11	0.748	0.77	0.28;2.15	0.625	0.80	0.36;1.77	0.582	0.71	0.28;1.78	0.466
Locorregional + distance	1.16	0.61;2.22	0.651	1.08	0.53;2.19	0.830	1.03	0.58;1.83	0.929	1.11	0.61;2.02	0.736
Line of therapy 2 or above	1.26	0.68;2.34	0.470	1.21	0.60;2.41	0.596	1.94	1.09;3.46	0.025	1.71	0.93;3.16	0.084
Multivariate analysis*	***HR***	***95% IC***	***P value***	***HR***	***95% IC***	***P value***	***HR***	***95% IC***	***P value***	***HR***	***95% IC***	***P value***
Age				0.99	0.96;1.02	0.359	#	#	#	0.97	0.94;1.00	0.026
Serum albumin	0.96	0.93;1.00	0.052	0.96	0.93;1.00	0.082	#	#	#	0.94	0.90;0.99	0.016
Low skeletal muscle index	2.19	1.19;4.05	0.012	2.79	1.37;5.67	0.005	#	#	#	1.90	1.04;3.48	0.037
Platinum-refractory	1.74	0.92;3.30	0.090	1.73	0.84;3.56	0.138	#	#	#	3.31	1.40;7.83	0.006
Line of therapy 2 or above							#	#	#	0.68	0.30;1.53	0.349

OS, overall survival; PFS, progression free survival; BMI, body mass index; ICI, immune checkpoint inhibitors; Type of recurrence includes locorregional disease, distance disease and locorregional+distance disease; Line of therapy includes first *vs* second or above lines.

*Adjusted for the covariates with p-value <0.1 in the univariate analysis.

^#^Multivariate models for global PFS were not computed due to the small numbers of patients in the no-event group.

**Figure 1:**
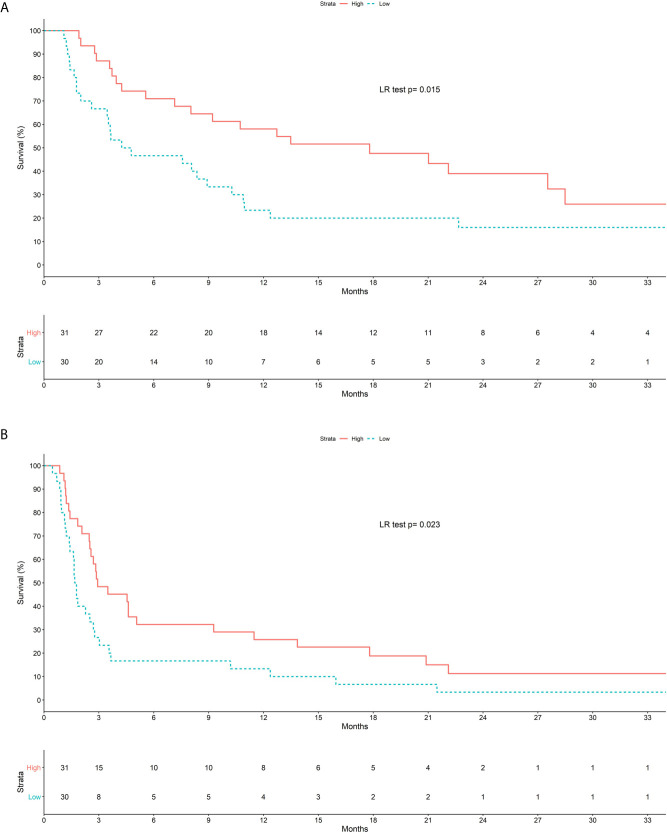
Kaplan-Meier survival curves according to baseline SMI. **(A)** Overall survival. **(B)** Progression Free Survival.

Patients with low SMI had shorter OS (HR, 2.06; 95% CI, 1.14–3.73; p = 0.017) (**Figure 1**) and 1-year OS rate (HR, 2.64; 95% CI, 1.33–5.23; p = 0.005) in the univariate analysis. Low SMI was also associated with global PFS (global PFS HR, 1.84; 95% CI, 1.08–3.12; p = 0.025, and 1-year PFS rate HR, 1.83; 95% CI, 1.01–3.23; p = 0.036) among other factors such as age (PFS HR, 0.96; 95% CI, 0.94–0.99; p = 0.002), baseline albumin (PFS HR, 0.95; 95% CI, 0.91–0.99; p = 0.027), platinum-refractory (PFS HR, 3.04; 95% CI, 1.67–5.56; p = <0.001), and any number of prior lines for R/M disease (PFS HR, 1.94; 95% CI, 1.09–3.46; p = 0.025). The association was maintained at 1-year PFS, although the number of prior lines showed only a trend (PFS HR, 1.71; 95% CI, 0.93–3.16; p = 0.084). One-year PFS showed a clear association with age (PFS HR, 0.97; 95% CI, 0.94–1.00; p = 0.036), serum albumin (PFS HR, 0.94; 95% CI, 0.90–0.99; p = 0.025), low SMI (PFS HR, 2.53; 95% CI, 1.19–5.37; p = 0.015), and patients who received platinum within 6 months prior to ICI (PFS HR, 3.57; 95% CI, 1.50–8.51; p = 0.004).

The multivariate analyses adjusted for serum albumin, baseline SMI, and platinum-refractory confirmed low SMI as an independent predictor for OS (HR, 2.19; 95% CI, 1.19–4.05; p = 0.012) and 1-year survival (HR, 2.79; 95% CI, 1.37–5.67; p = 0.005) adjusting this analysis also for age. Type of recurrence prior ICI initiation and BMI were not included in the multivariate analysis because it did not show association for survival or PFS in the univariate analysis.

Similar results were found using previously published cut points for sarcopenia (28) for OS but not for PFS. These results showed that sarcopenia was an independent factor for OS (HR, 2.06; 95% CI, 1.01–4.23; p = 0.048) after adjusting by the same covariates than the analysis performed for low SMI. This analysis is provided as **Supplementary Information** (**Table 2S** and **Figure 1S**).

We sought to determine whether different BMIs were associated with any of the abovementioned outcomes. There were no statistically significant differences in OS and PFS, when examining overweight or obese patients (BMI ≥ 25 kg/m^2^) compared with patients with normal BMI.

### Treatment Toxicity

IrAEs occurred in 29 (47.5%) patients mainly in those treated with anti-PDL1 plus IOA (n = 15; 80%) and treated with anti-PD1 plus chemotherapy (n = 2; 66.7%). Thyroiditis, skin, and liver alterations were the most common IrAEs, and the vast majority was grade 1 or grade 2. Only seven patients developed grade 3 or above toxicity, three of them with low SMI.

There was no association with low SMI and IrAEs of any grade (OR, 0.56; 95% CI, 0.20–1.54; p = 0.261).

Different factors were examined to determine their effect in the development of IrAEs. Patients with BMI ≤18.5 kg/m^2^ (OR, 0.09; 95% CI, 0.00–0.63; p = 0.012), the presence of distance metastasis (OR, 4.93; 95% CI, 1.01–30.4, p = 0.048), and those patients platinum-refractory (OR, 0.33; 95% CI, 0.11–0.94, p = 0.037) were associated with toxicity of any grade. In the multivariate analysis, only being refractory to platinum (OR, 2.88; 95% CI, 1.05–8.98, p = 0.050) was a predictive factor of IrAEs occurrence. The presence of distant metastasis was not included in the multivariate analysis because only three patients with distant metastasis did not develop IrAEs.

## Discussion

Immunotherapy is significantly changing the therapeutic landscape for R/M HNSCC (4, 5). Its clinical efficacy varied among HNSCC patients, and there is a lack of accurate and effective predictive biomarkers. Low SMI is frequently encountered in HNSCC patients (10, 16). However, whether low SMI can be used as a predictive biomarker for ICI remains unknown, and the clinical data regarding the association between SMI and ICI efficacy are quite limited. To the best of our knowledge, this is the first study to assess the association between SMI and clinical outcomes of R/M HNSCC patients undergoing ICI therapy.

In our study, low SMI was confirmed to be an independent factor for reduced OS and 1-year survival after adjusting the model for relevant factors associated with clinical outcomes in HNSCC. These findings are in agreement with other studies performed in melanoma and lung cancer (19, 31). We did not assess mortality specific for cancer as only two patients died from another cause different from the primary cancer. Important variables such as age, serum albumin, refractoriness to platinum or the number of lines of therapy prior to ICI therapy are well-known predictive factors. However, body composition is often overlooked in clinical practice. BMI is not a good indicator of body composition as elevated BMI may hide a distribution of low muscle mass increasing the risk for adverse outcomes (19, 32). Moreover, muscle has been shown to be one of the strongest parameters associated with mortality in cancer patients (33) even when weight and BMI are included in the analysis.

There are numerous cut points values published for sarcopenia although none of them is yet definitive. Some of these cut points used OS as the outcome according to the SMI (27, 30). We have chosen Martin et al. (30) as their population is a large sample with advanced stage and includes all BMIs. Moreover, these cut points have been used in many previous publications, so readers can compare our results. As our cohort is a slightly distinct population, we also chose the median L3 SMI cut point to evaluate SMI as a predictive biomarker in R/M HNSCC.

Although the mechanism by which reduced SMI has a negative effect on the clinical efficacy of ICI remains unclear. New evidence shows that skeletal muscle cells, as an endocrine organ, may secrete specific cytokines that regulate immunity. These myokines are involved in modulating the immune response (34). Thus, a reduction in muscle mass may have a deleterious effect on the anti-tumor response mediated by the immune system, following in immunosuppression (35). A decrease in myokines due to the loss of muscle mass could suppress tumor response to ICI, resulting in the immune escape of tumor cells (36, 37). Inflammation also plays an important role in the loss of muscle mass (38). All these factors may contribute to the impairment of the antitumor immune response to ICI in HNSCC.

We did not find any statistically significant associations between BMI and clinical outcomes to ICI. Young et al. (19) identified trends toward worse outcomes in patients with high BMI and low muscle mass in patients with melanoma treated with anti-PD1. However, we included only six patients with BMI ≥ 25 kg/m^2^ and low SMI.

Compared with traditional treatments (chemotherapy or radiotherapy), the incidence of toxicity in HNSCC patients treated with ICI has been reduced. We explored the effect of low SMI on the incidence of adverse event related to ICI in HNSCC patients, finding that low SMI was not significantly associated with the incidence of IrAEs. Evidence suggests that the incidence of IrAEs of any grade is associated with improved clinical outcomes (39). Unfortunately, subgroups analysis could not be further performed because of insufficient data.

Our study has some limitations that should be addressed. The main ones are the retrospective design of the study and the limited number of patients. Moreover, the CT imaging analysis was limited by the data availability; indeed, the acquisition protocol was planned according to the presence of previous examination. Finally, we did not take into account the type of ICI therapy alone or in combination.

In conclusion, our finding shows that baseline SMI is an independent factor for survival R/M HNSCC treated with ICI. SMI is not associated with the onset of IrAEs. Further prospective research is needed to confirm the role of body composition as a predictive biomarker in ICI treatment and how SMI can affect drug-specific and organ-specific adverse events caused by ICI in HNSCC patients.

## Data Availability Statement

The datasets presented in this study can be found in online repositories. The names of the repository/repositories and accession number(s) can be found below: IDIBELL repository (http://diposit.ub.edu/dspace/handle/2445/172899).

## Ethics Statement

The studies involving human participants were reviewed and approved by Hospital Universitari de Bellvitge Ethics Committee for Clinical Research (PR302/18). The patients/participants provided their written informed consent to participate in this study.

## Author Contributions

LA, MP, MT, RM, and VB were actively involved in the design of the stud writing the manuscript. LA, MP, MS, NV, AT, and LR were involved in the collection of data. NP and LA analyzed the data and all authors interpreted the data reviewed the manuscript. RM and VB supervised the study. All authors contributed to the article and approved the submitted version.

## Funding

With the support of the “Acció instrumental d’intensificació de professionals de la salut” (grant number SLT008/18/00047) of the Department of Health of the Government of Catalonia. We thank CERCA Programme/Generalitat de Catalunya for institutional support.

## Conflict of Interest

The authors declare that the research was conducted in the absence of any commercial or financial relationships that could be construed as a potential conflict of interest.
