# Supplementary material for: Predictive Value of Skeletal Muscle Mass in Recurrent/Metastatic Head and Neck Squamous Cell Carcinoma Patients Treated With Immune Checkpoint Inhibitors
Source: Front Oncol. 2021 Jun 25;11:699668. doi: 10.3389/fonc.2021.699668 (PMC8267860; doi:10.3389/fonc.2021.699668)
Supplement: Supplementary file 1 [file DataSheet_1.docx]

Supplementary Material

# Supplementary Figures and Tables

Table 1S. Patient baseline characteristics (overall and according to the presence of sarcopenia) (n=61)

|  | Overall  (n=61) | Sarcopenia  (n=41) | No sarcopenia (n=20) | p-overall |
| --- | --- | --- | --- | --- |
| Age, years |  |  |  |  |
| Mean (SD) | 57.7 (9.62) | 57.2 (9.94) | 58.7 (9.10) | 0.561 |
| Median (range) | 59.0 (23-78) | 59.0 (23-70) | 59.5 (35-78) | 0.753 |
| Male, n (%) | 52 (85.2) | 35 (85.4) | 17 (85.0) | 1.000 |
| Smoking status, n (%) |  |  |  | 0.180 |
| Current | 28 (45.9) | 22 (53.7) | 6 (30.0) |  |
| Former* | 26 (42.6) | 14 (34.1) | 12 (60.0) |  |
| Never | 7 (11.5) | 5 (12.2) | 2 (10.0) |  |
| Location, n (%) |  |  |  | 0.556 |
| Oral cavity | 21 (34.4) | 14 (34.1) | 7 (35.0) |  |
| Hypopharynx | 8 (13.1) | 7 (17.1) | 1 (5.0) |  |
| Larynx | 19 (31.3) | 11 (26.8) | 8 (40.0) |  |
| Oropharynx** | 13 (21.3) | 9 (22.0) | 4 (20.0) |  |
| Type of recurrence |  |  |  | 0.254 |
| Locoregional | 23 (37.7) | 14 (34.1) | 9 (45.0) |  |
| Distance | 10 (16.4) | 9 (22.0) | 1 (5.0) |  |
| Locoregional + distance | 28 (45.9) | 18 (43.9) | 10 (50.0) |  |
| Line of therapy, n (%) |  |  |  | 0.871 |
| First | 22 (36.1) | 14 (34.1) | 8 (40.0) |  |
| Second or above | 39 (63.9) | 27 (65.9) | 12 (60.0) |  |
| Type of ICI therapy, n (%) |  |  |  | 0.752 |
| AntiPD1 | 8 (13.1) | 6 (14.6) | 2 (10.0) |  |
| AntiPD1+virus | 1 (1.64) | 1 (2.4) | 0 (0.0) |  |
| AntiPD1 + chemotherapy | 3 (4.92) | 3 (7.3) | 0 (0.0) |  |
| AntiPDL1 | 12 (19.7) | 9 (22.0) | 3 (15.0) |  |
| AntiPDL1+antiCTLA4 | 22 (36.1) | 13 (31.7) | 9 (45.0) |  |
| AntiPDL1+IOA | 15 (24.6) | 9 (22.0) | 6 (30.0) |  |
| ECOG-PS, n (%) |  |  |  | 0.032 |
| 0 | 1 (1.64) | 0 (0) | 1 (5.00) |  |
| 1 | 58 (95.1) | 41 (100) | 17 (85.0) |  |
| 2 | 2 (3.28) | 0 (0) | 2 (10.0) |  |
| Platinum within 6 months of ICI,n (%) | 36 (59.0) | 25 (61.0) | 11 (55.0) | 0.866 |
| Weight, kg |  |  |  |  |
| Mean (SD) | 67.3 (15.0) | 66.2 (15.4) | 69.5 (14.2) | 0.413 |
| Median [Q1; Q3] | 65.2 [54.3;79.0] | 62.9 [54.0;77.8] | 72.2 [59.8;80.8] | 0.303 |
| BMI, kg/m^2^ |  |  |  |  |
| Mean (SD) | 23.8 (4.56) | 23.4 (4.73) | 24.7 (4.16) | 0.260 |
| Median (range) | 23.6 (15.8-34.7) | 22.9 (15.8-34.7) | 24.7 (17.7-32.1) | 0.208 |
| BMI categorized, kg/m^2^ |  |  |  | 0.869 |
| Underweight (<18.5) | 9 (14.8) | 7 (17.1) | 2 (10.0) |  |
| Normal (18.5 – 25) | 26 (42.6) | 17 (41.5) | 9 (45.0) |  |
| Overweight /obese (>25) | 26 (42.6) | 17 (41.5) | 9 (45.0) |  |
| Albumin, g/L |  |  |  |  |
| Mean (SD) | 42.9 (60.3) | 42.0 (6.87) | 44.9 (3.23) | 0.032 |
| Median [Q1; Q3] | 44.0 [41.0;46.0] | 43.0 [41.0;45.0] | 45.0 [42.0;47.2] | 0.025 |
| SMI, cm^2^/m^2^ |  |  |  |  |
| Mean (SD) | 43.6 (7.75) | 40.1 (5.79) | 50.8 (6.17) | <0.001 |
| Median [Q1; Q3] | 42.0 [37.5;48.6] | 39.0 [36.1;42.0] | 48.8 [46.2;55.0] | <0.001 |
| TATI, cm^2^/m^2^ |  |  |  |  |
| Mean (SD) | 91.4 (53.3) | 92.6 (54.9) | 88.9 (51.1) | 0.798 |
| Median [Q1; Q3] | 98.8 [49.4;118] | 92.3 [49.9;138] | 99.7 [56.1;112] | 0.794 |

**Ex-smoker defined as no cigarettes for more than 6 months before diagnosis*

*ICI, immune checkpoint inhibitor; ECOG-PS, Eastern Cooperative Oncology Group Performance Status; BMI, body mass index; SMI, skeletal muscle index; TATI, total adipose tissue index*

*** 3 of them HPV-related*

Table 2S. Univariate and multivariate analysis examining OS, one-year survival, global PFS and one-year PFS in association with sarcopenia (n=61).

|  | SURVIVAL | | | | | | PROGRESION FREE SURVIVAL | | | | | |
| --- | --- | --- | --- | --- | --- | --- | --- | --- | --- | --- | --- | --- |
|  | **Overall survival** | | | **1-year survival** | | | **Global PFS** | | | **1-year PFS** | | |
| Univariate analysis | ***HR*** | ***95% IC*** | ***P value*** | ***HR*** | ***95% IC*** | ***P value*** | ***HR*** | ***95% IC*** | ***P value*** | ***HR*** | ***95% IC*** | ***P value*** |
| BMI | 0.97 | 0.91;1.04 | 0.432 | 0.96 | 0.89;1.03 | 0.232 | 0.96 | 0.91;1.02 | 0.237 | 0.96 | 0.90;1.03 | 0.235 |
| Age | 0.98 | 0.96;1.01 | 0.177 | 0.98 | 0.95;1.00 | 0.077 | 0.96 | 0.94;0.99 | 0.002 | 0.96 | 0.94;0.99 | 0.002 |
| Serum albumin | 0.97 | 0.93;1.00 | 0.056 | 0.97 | 0.93;1.01 | 0.112 | 0.95 | 0.91;0.99 | 0.027 | 0.95 | 0.90;0.99 | 0.024 |
| Sarcopenia | 2.21 | 1.11;4.37 | 0.023 | 2.32 | 1.06;5.10 | 0.036 | 1.40 | 0.79;2.46 | 0.248 | 1.49 | 0.82;2.72 | 0.192 |
| Platinum-refractory | 1.76 | 0.94;3.28 | 0.075 | 1.85 | 0.91;3.78 | 0.089 | 3.04 | 1.67;5.56 | <0.001 | 2.95 | 1.57;5.55 | 0.001 |
| Type of recurrence |  |  |  |  |  |  |  |  |  |  |  |  |
| Distance | 0.86 | 0.35;2.11 | 0.748 | 0.77 | 0.28;2.15 | 0.625 | 0.80 | 0.36;1.77 | 0.582 | 0.71 | 0.28;1.78 | 0.466 |
| Locorregional + distance | 1.16 | 0.61;2.22 | 0.651 | 1.08 | 0.53;2.19 | 0.830 | 1.03 | 0.58;1.83 | 0.929 | 1.11 | 0.61;2.02 | 0.736 |
| Line of therapy  2 or above | 1.26 | 0.68;2.34 | 0.470 | 1.21 | 0.60;2.41 | 0.596 | 1.94 | 1.09;3.46 | 0.025 | 1.71 | 0.93;3.16 | 0.084 |
| Multivariate analysis | ***HR*** | ***95% IC*** | ***P value*** | ***HR*** | ***95% IC*** | ***P value*** | ***HR*** | ***95% IC*** | ***P value*** | ***HR*** | ***95% IC*** | ***P value*** |
| BMI | 1.20 | 0.45;3.19 | 0.717 | 1.40 | 0.51;3.85 | 0.517 | # | # | # | 1.08 | 0.44;2.64 | 0.861 |
| Age | 0.98 | 0.95;1.01 | 0.225 | 0.98 | 0.95;1.01 | 0.138 | # | # | # | 0.96 | 0.94;0.99 | 0.010 |
| Serum albumin | 0.97 | 0.94;1.01 | 0.179 | 0.97 | 0.93;1.02 | 0.226 | # | # | # | 0.95 | 0.90;1.00 | 0.044 |
| Sarcopenia | 2.06 | 1.01;4.23 | 0.048 | 2.05 | 0.90;4.67 | 0.087 | # | # | # | 1.50 | 0.78;2.87 | 0.222 |
| Platinum-refractory | 1.97 | 0.81;4.76 | 0.133 | 2.04 | 0.76;5.50 | 0.158 | # | # | # | 3.17 | 1.33;7.57 | 0.009 |
| Line of therapy  2 or above | 0.63 | 0.26;1.52 | 0.301 | 0.51 | 0.19;1.39 | 0.189 | # | # | # | 0.61 | 0.25;1.46 | 0.264 |

*OS, overall survival; PFS, progression free survival; BMI, body mass index; SMI, skeletal muscle index; ICI: immune checkpoint inhibitors; Type of recurrence includes locorregional disease, distance disease and locorregional+distance disease; Line of therapy includes first vs second or above lines.*

**Adjusted for age, serum albumin, baseline SMI, BMI, line of therapy and platinum-refractory.*

*# Multivariate models for global PFS were not computed due to the small numbers of patients in the no-event group.*

## Supplementary Figures

Figure 1S. Kaplan-Meier survival curves according to sarcopenia. (A) Overall survival. (B) Progression Free Survival

(A)


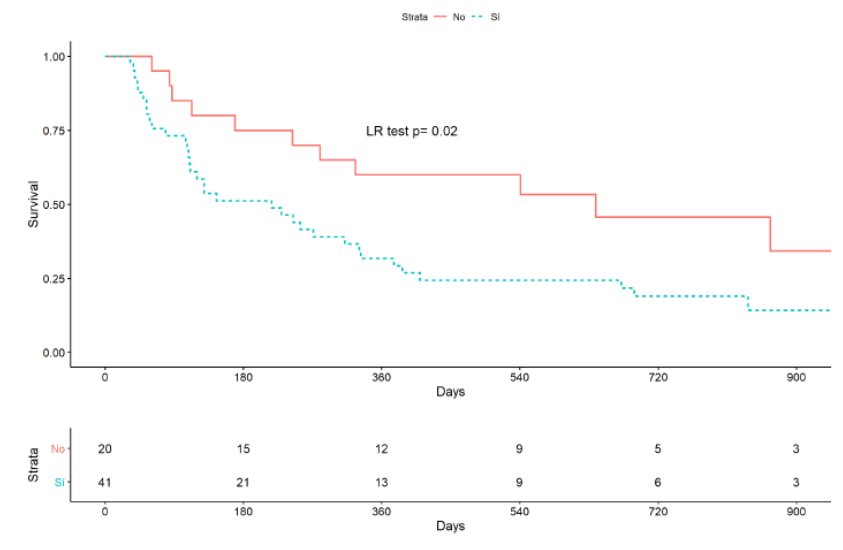


(B)


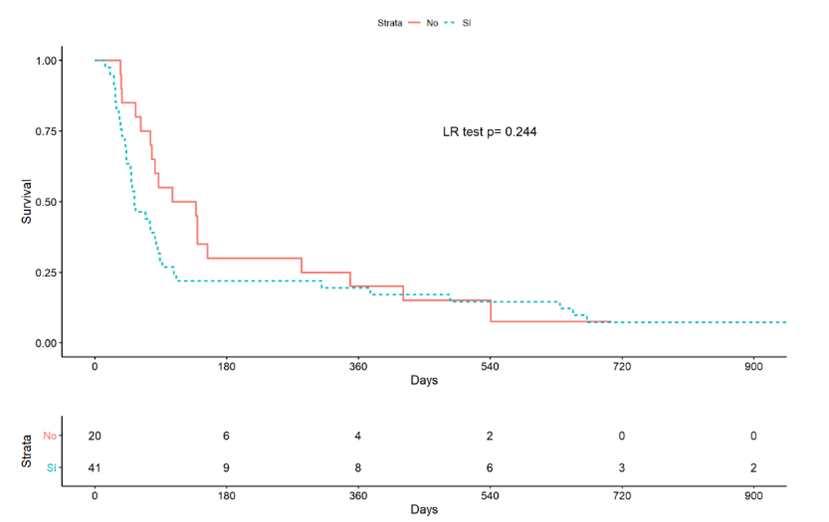


**
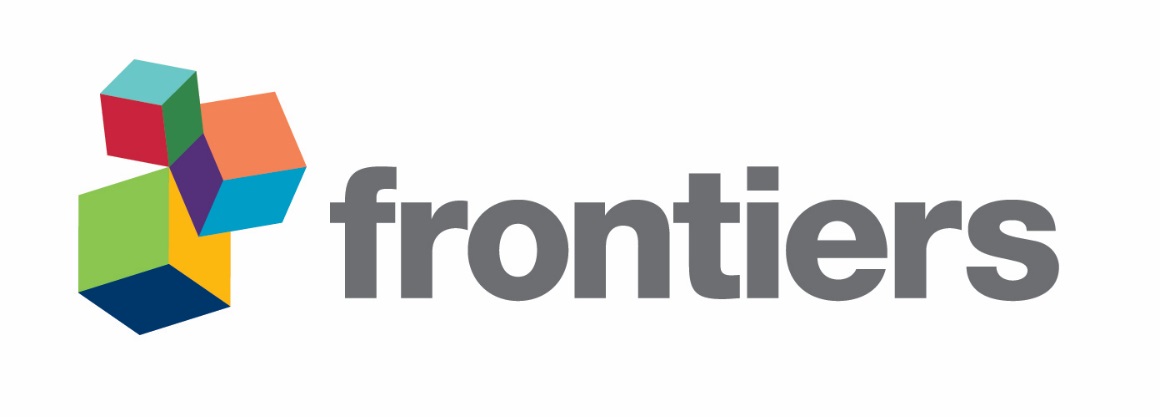
**
